# Increased snowfall weakens complementarity of summer water use by different plant functional groups

**DOI:** 10.1002/ece3.5058

**Published:** 2019-03-15

**Authors:** Yonggang Chi, Lei Zhou, Qingpeng Yang, Shao‐peng Li, Shuxia Zheng

**Affiliations:** ^1^ College of Geography and Environmental Sciences Zhejiang Normal University Jinhua China; ^2^ State Key Laboratory of Vegetation and Environmental Change, Institute of Botany Chinese Academy of Sciences Beijing China; ^3^ School of Biological Sciences Georgia Institute of Technology Atlanta Georgia; ^4^ Key Laboratory of Ecosystem Network Observation and Modelling, Institute of Geographic Sciences and Natural Resources Research Chinese Academy of Sciences Beijing China; ^5^ Key Laboratory of Forest Ecology and Management, Institute of Applied Ecology Chinese Academy of Sciences Shenyang China

**Keywords:** hydrogen stable isotope signature (δ*D*), Inner Mongolia grassland, oxygen stable isotope signature (δ^18^O), rainfall, snowfall, water use partitioning

## Abstract

Winter snowfall is an important water source for plants during summer in semiarid regions. Snow, rain, soil water, and plant water were sampled for hydrogen and oxygen stable isotopes analyses under control and increased snowfall conditions in the temperate steppe of Inner Mongolia, China. Our study showed that the snowfall contribution to plant water uptake continued throughout the growing season and was detectable even in the late growing season. Snowfall versus rainfall accounted for 30% and 70%, respectively, of the water source for plants, on the basis of hydrogen stable isotope signature (δ*D*) analysis, and accounted for 12% and 88%, respectively, on the basis of oxygen stable isotope signature (δ^18^O) analysis. Water use partitioning between topsoil and subsoil was found among species with different rooting depths. Increased snowfall weakened complementarity of plant water use during summer. Our study provides insights into the relationships between precipitation regimes and species interactions in semiarid regions.

## 1 INTRODUCTION

Precipitation is an essential factor that largely controls plant community structure and ecosystem functioning in semiarid regions (Bai et al., 2008; Huston, 2012). Rainfall, the type of precipitation that falls mostly in the growing season, directly regulates ecosystem processes in the growing season by changing soil water availability (Hu, Hopping, Bump, Kang, & Klein, 2013). However, snowfall, the type of precipitation that falls in the nongrowing season, could indirectly mediate ecosystem processes in the growing season by altering, for example, winter near‐surface soil temperature **(**Brown & DeGaetano, 2011 **)**, microbial activity and organic matter decomposition during winter (Buckeridge & Grogan, 2008), and the length of the nongrowing season **(**Jonas, Rixen, Sturm, & Stoeckli, 2008; Wipf, Rixen, & Mulder, 2006 **)**. Plant water use strategy in semiarid region is tied heavily to rainfall during the growing season and snowfall during the nongrowing season (Chesson et al., 2004). Therefore, disentangling the relative contributions of rainfall versus snowfall to plant water use is essential for gaining a thorough understanding of how changed precipitation regimes, projected under future climate change scenarios, will influence semiarid regions.

Niche complementarity postulates that coexisting plant species requires differentiation along niche axes, such as light, soil moisture, and available nutrients (Schwinning & Kelly, 2013). In semiarid region, seasonality of precipitation allows for many axes of niche differentiation, particularly with regard to plant water uptake strategies (Chesson et al., 2004). In theory, the complementarity of plant water uptake might be achieved by the intrinsic stratification in their rooting depth (Nippert & Holdo, 2015). However, roots have the ability to shift among different water sources to exploit water resources under fluctuating environments (Ehleringer & Dawson, 1992). Although precipitation is an important resource related to plant performance, there has been a lack of direct measurements that can be used to assess water use partitioning among species (Mommer et al., 2010).

Hydrogen and oxygen stable isotopes analysis has been used to directly estimate the water sources of plants (e.g., water from different soil depths, stream water vs. groundwater, snowmelt vs. rain) (Barnard, Bello, Gilgen, & Buchmann, 2006; Penna et al., 2018; Xu et al., 2011). Specifically, water sources of co‐occurring species can be identified by comparing the natural abundance of stable isotopes in plant water and in potential water sources (Phillips & Gregg, 2003). Proportional contributions of water sources to the plants can then be estimated by two‐ or three‐layer mixing models (Dawson, 1993), although which have a limitation in determining the ranges of possible contributions from different water sources (Wang, Song, Han, Zhang, & Liu, 2010). Several studies have successfully used the natural abundance of stable isotopes to determine the proportional use of different water sources (Asbjornsen, Mora, & Helmers, 2007) and to quantitatively analyze hydrological processes (Williams & Ehleringer, 2000).

The semiarid steppe in northern China, with an area of 313 million hm^2^ and a plant species richness of 2,300, plays an important role in serving the economy and well‐being of people residing in this region **(**Kang, Han, Zhang, & Sun, 2007 **)**. In this region, the summer rainfall mainly recharges the topsoil layers, while the winter snowfall recharges the subsoil layers (Yang, Auerswald, Bai, & Han, 2011). Soil water levels typically begin to decline between spring and midsummer before the arrival of rainfall (Yang et al., 2011). Therefore, an increase in snowfall may increase the amount of soil water during the early growing season, alleviating some of the water stress that plants experience before the arrival of rainfall (Hu et al., 2013). Observational studies and model simulations for this region predict an increase in both the mean and extreme “snow disaster” snowfall (O'Gorman, 2014; Peng, Piao, Ciais, Fang, & Wang, 2010). Here, our objectives were (a) to quantitatively analyze the relative contributions of snowfall versus rainfall to plant water uptake and (b) to evaluate the effects of increased snowfall on water use partitioning by different plant functional groups.

## 2 MATERIALS AND METHODS

### 2.1 Study area and experimental design

The study was conducted at the Inner Mongolia Grassland Ecosystem Research Station (IMGERS; 43°38′N, 116°42′E and 1,250 m a.s.l.), which is located in the temperate steppe in Inner Mongolia, China (Supporting Information Figure S1). The long‐term (2001–2015) mean annual temperature in the study area is 2.55°C, with mean monthly temperatures ranging from −16.92°C in January to 19.25°C in July. The long‐term (2001–2015) mean annual precipitation is 305.2 mm, with 20.6 mm falling as snow and 284.6 mm falling as rain, that is, snowfall represented 7% of the annual precipitation.

The soil texture is dark chestnut with loamy sand (Bai et al., 2010). The soils are characterized as slightly alkaline and nutrient poor, with pH values of 7.22 ± 0.03, bulk density of 1.43 ± 0.03 g/cm^3^, and soil total carbon and nitrogen contents of 7.73 ± 0.25 and 0.91 ± 0.02 g/kg, respectively, in the top 10‐cm layer. The aboveground biomass (AGB) in this semiarid region is low (184 g/m^2^). The plant communities are dominated by *Stipa grandis *P. Smirn. (shallow‐rooted species), *Agropyron cristatum* (L.) Garrtn. (medium‐rooted species), and *Artemisia frigida* Willd. (deep‐rooted species), and the corresponding coverage are 6%, 9%, and 22%, respectively. Previous studies reported that the roots of *Stipa grandis *P. Smirn. only extend to 15 cm depth (Wang et al., 2016), that of *Agropyron cristatum* (L.) Garrtn. to 25 cm depth (Yang et al., 2011), and that of *Artemisia frigida* Willd. to 65 cm depth (Liang et al., 2009). Therefore, *Stipa grandis *P. Smirn. are defined as shallow‐rooted species, *Agropyron cristatum* (L.) Garrtn. as medium‐rooted species, and *Artemisia frigida* Willd. as deep‐rooted species in current study.

Snow fences were constructed to create snowdrifts and to manipulate snow depth on the leeward side of the fences (Griffith & Loik, 2010). The plastic mesh fencing was 50% porosity and 1.2 m high and was oriented approximately south to north, which is generally perpendicular to the prevailing wind direction. The plots for the soil and plant collection were selected under two different snow depth regimes (i.e., the control and the increased snowfall) with three replicates for each treatment. There were a total of six plots (i.e., 3 plots per treatment × 2 treatments = 6 plots). The increased snowfall plots were established approximately 4 m downwind of the fences, and the control plots were established approximately 28 m downwind of the fences where the snow depth was unaffected by the presence of the fence. The size of the plots is 2 × 3 m^2^.

The maximum snow depth was 25.0 ± 1.2 and 122.0 ± 17.4 cm, respectively, for the control and increased snowfall plots. Measurements of snow depth were conducted using poles that were pushed through the snow to the soil surface on 16 January 2016, which corresponded to the annual peak snowpack depth (Supporting Information Figure S1). The snow water equivalent was a product of snow depth multiplied by snow bulk density (0.13 g/cm^3^ for northern China, Mo, Dai, Fan, Che, & Hong, 2016). In control plots, the precipitation in 2016 was 275.6 mm, with 32.5 mm falling as snow and 243.1 mm falling as rain, that is, snowfall represented 12% of the annual precipitation. In increased snowfall plots, the precipitation in 2016 was 401.7 mm, with 158.6 mm falling as snow and 243.1 mm falling as rain, that is, snowfall represented 40% of the annual precipitation. The amount of snowfall in control plots was within the range of a normal year, and the amount of snowfall in increased snowfall plots was within the range of a large “snow disaster” snowstorm (Peng et al., 2010).

The snow in the control plots completely melted by March 15, and the snow in the increased snowfall plots completely melted by March 23. The fences were removed during the growing season to avoid the unwanted effects of shading and wind disruption.

### 2.2 Field sampling and measurements

When a precipitation event occurred, precipitation samples were collected at the meteorological observation field site approximately 100 m from the snow fences. Snow samples were collected using large PVC pipes that were fitted with closed bottoms. Subsamples of snow were sealed in 100 ml bottles until they melt in the laboratory. Snow water samples were immediately enclosed in 8‐ml airtight glass vials and stored in a refrigerator at 4°C. Three precipitation samples were taken per precipitation event.

Rain samples were collected using Nalgene bottles that were fitted with funnels. Mineral oil was added to the rainwater collectors to prevent evaporative enrichment of isotopes. Rainwater samples were immediately sealed in 8‐ml airtight glass vials and stored in a refrigerator at 4°C.

Soil and plant samples were collected synchronously in different community growth periods, that is, early growing season (April 20 and May 10), middle growing season (June 14 and July 15), and late growing season (August 10 and September 19). The soil samples were taken in the vicinity of the plants which were selected. Soil samples were collected by taking two 7‐cm‐diameter soil cores at depth increments of 0–10, 10–20, 20–40, 40–60, and 60–100 cm at each plot. Two cores from each plot were mixed in situ to form one composite sample. There was one replicate for each soil depth at each plot. Soil samples were immediately sealed in 8‐ml airtight glass vials and stored in a refrigerator at 4°C. A total of 180 soil samples each year were taken and analyzed for hydrogen and oxygen stable isotope (i.e., 1 soil sample per soil depth × 5 soil depths per plot × 3 plots per treatment × 2 treatments per month × 6 months per year = 180 soil samples per year).

The plant samples were collected from the three species with different rooting depths (i.e., shallow‐rooted species *Stipa grandis *P. Smirn., medium‐rooted species *Agropyron cristatum* (L.) Garrtn., and deep‐rooted species *Artemisia frigida* Willd.). Plant samples were collected at the root crown, often at or just below the soil surface (Barnard et al., 2006). For each species, the root crowns from at least 10 individuals were collected as one replicate, enclosed in 8‐ml airtight glass vials and stored in a refrigerator at 4°C. There was one replicate for each plant species at each plot. A total of 108 plant samples each year were taken and analyzed for hydrogen and oxygen stable isotope (i.e., 1 plant sample per species × 3 species per plot × 3 plots per treatment × 2 treatments per month × 6 months per year = 108 plant samples per year). The sampling date for soil and plant was selected when there was no rainfall for at least 3 days, and the percolating movement of soil water was stable (Wang et al., 2010). The sampling time was chosen between 7:00 and 9:00 a.m. when evapotranspiration was low.

Hydrogen and oxygen stable isotopes analysis was performed in the Stable Isotope Laboratory for Ecological and Environmental Research, Chinese Academy of Forestry. All water samples from the snow, rain, soil, and plants were analyzed for hydrogen and oxygen stable isotopes using the temperature conversion elemental analysis (TC/EA) method (Finnigan MAT253, Thermo Fisher Scientific, USA). The precision of hydrogen stable isotope signature (δ*D*, ‰) is ±1‰, and the precision of oxygen stable isotope signature (δ^18^O, ‰) is 0.1%. Soil water and plant water were extracted using the cryogenic vacuum distillation method (Ehleringer, Phillips, Schuster, & Sandquist, 1991). δ*D* was calculated using the hydrogen stable isotopic composition:

(1) $$\delta D=\left(\frac{R_{\text{sample}}}{R_{\text{standard}}}-1\right)\times 1000‰$$

where *R*_sample_ and *R*_standard_ are the hydrogen stable isotopic compositions (i.e., the D/H molar ratio) of the sample and the standard water (i.e., VSMOW, Vienna Standard Mean Ocean Water), respectively (Phillips & Gregg, 2003). Similarly, δ^18^O was calculated using the oxygen stable isotopic composition.

The soil water content (SWC) was measured at depth increments of 0–10, 10–20, 20–40, 40–60, and 60–100 cm in early, middle, and late growing season. The soil samples from each layer were placed into aluminum boxes and dried in an oven at 105°C for 24 hr. The AGB and belowground biomass (BGB) were sampled on August 10, which corresponded to the annual peak in standing biomass (Bai et al., 2010). AGB was determined as the weight of the aboveground plant material in each 1 × 1 m quadrat after oven drying at 65°C for 48 hr. BGB was sampled at depth increments of 0–10, 10–20, 20–40, 40–60, and 60–100 cm by taking two 7‐cm‐diameter soil cores. In our current study, root biomass was sampled by plots and soil depths, not per species, because the identification of roots among species is very difficult in the field. Roots were cleaned by placing them under running water over a 1‐mm screen, and the cleaned roots were oven dried at 65°C for 48 hr and then weighed.

### 2.3 Statistical analyses

The rainfall contribution to soil water (*f*_rainfall to soil_) was determined by using two‐source linear mixing models on the basis of δ*D* analysis (Phillips & Gregg, 2003):

(2) $$f_{\text{rain}\;\text{to}\;\text{soil}}=\frac{\delta D_{\text{soil}}-\delta D_{\text{snow}}}{\delta D_{\text{rain}}-\delta D_{\text{snow}}}\times 100\%$$

where δ*D*_soil_ is the δ*D* value of the soil water, δ*D*_snow_ is the δ*D* value of the snowfall, and δ*D*_rain_ is the mean δ*D* value of the rainfall between the two soil samplings. The sleet events represent only 2.6% of all precipitation events in China (Chen, Liu, & Song, 2014). In view of the sleet/precipitation ratio is considerably low in every season, we do not take the magnitude of rainfall/snowfall events into account. In particular, the δ*D*_rain_ on April 20 was the mean δ*D* value of the rainfall between April 1 and April 20, that is, from the onset of the growing season to the first sampling date. The value of *f* is set to 0 when the δ*D*_soil_ is lower than the δ*D*_snow_, and the value of *f* is set to 1 when the δ*D*_soil_ is higher than the δ*D*_rain _(Cheng et al., 2006; Yang et al., 2011). Similarly, the analysis with δ^18^O was also run to determine *f*_rainfall to soil_. The snowfall contribution to soil water (*f*_snowfall to soil_) on the basis of δ*D* and δ^18^O analysis was calculated as:

(3) $$f_{\text{snow}\;\text{to}\;\text{soil}}=1-f_{\text{rain}\;\text{to}\;\text{soil}}$$

The rainfall contribution to plant water uptake (*f*_rainfall to plant_) was determined by using two‐source linear mixing models on the basis of δ*D* analysis (Phillips & Gregg, 2003):

(4) $$f_{\text{rain to plant}}=\frac{\delta D_{\text{plant}}-\delta D_{\text{snow}}}{\delta D_{\text{rain}}-\delta D_{\text{snow}}}\times 100\%$$

where δ*D*_plant_ is the δ*D* value of the plant stem water. δ*D*_snow_ and δ*D*_rain_ are the two extreme ends of the water sources in terms of the lowest and highest δ*D* values. Similarly, the analysis with δ^18^O was also run to determine *f*_rainfall to plant_. The snowfall contribution to plant water uptake (*f*_snowfall to plant_) on the basis of δ*D* and δ^18^O analysis was calculated as:

(5) $$f_{\text{snow}\;\text{to}\;\text{plant}}=1-f_{\text{rain}\;\text{to}\;\text{plant}}$$

The direct inference approach was used to assess the main depth of root water uptake (Wang et al., 2010). Specifically, by directly comparing the δ*D* and δ^18^O between the soil water profile and the root crown, the depths of soil water with similar isotope values to plant water may indicate the main depth of root water uptake. The main depth of root water uptake was set to 0 when the plant water signal did not “overlap” with the soil water signal. This method assumed that the main depth of root water uptake only occurred in one depth interval. The coefficient of variation (CV) for the main depth of root water uptake among species represents the complementarity of water use (Kühsel & Blüthgen, 2015).

Repeated Measures ANOVA (RMANOVA) were used to examine increased snowfall and soil depth effects on δ*D*_soil_, δ^18^O_soil_, contribution of snowfall to soil water on the basis of δ*D* analysis, contribution of snowfall to soil water on the basis of δ^18^O analysis, and SWC over the growing seasons. Between‐subject effects were evaluated as increased snowfall and soil depth, and within‐subject effects were time‐of‐season. Similarly, RMANOVA were used to examine increased snowfall and plant species effects on δ*D*_plant_, δ^18^O_plant_, contribution of snowfall to plant water uptake on the basis of δ*D* analysis, contribution of snowfall to plant water uptake on the basis of δ^18^O analysis, the main depth of root water uptake on the basis of δ*D* analysis, and the main depth of root water uptake on the basis of δ^18^O analysis over the growing seasons. Between‐subject effects were evaluated as increased snowfall and plant species, and within‐subject effects were time‐of‐season. One‐way ANOVA was used to analyze the effects of increased snowfall on AGB, BGB, and CV for the main depth of root water uptake among species. All statistical analyses were conducted using SPSS (version 17.0, SPSS Inc., USA).

## 3 RESULTS

### 3.1 Hydrogen stable isotope in precipitation

**Figure 1:**
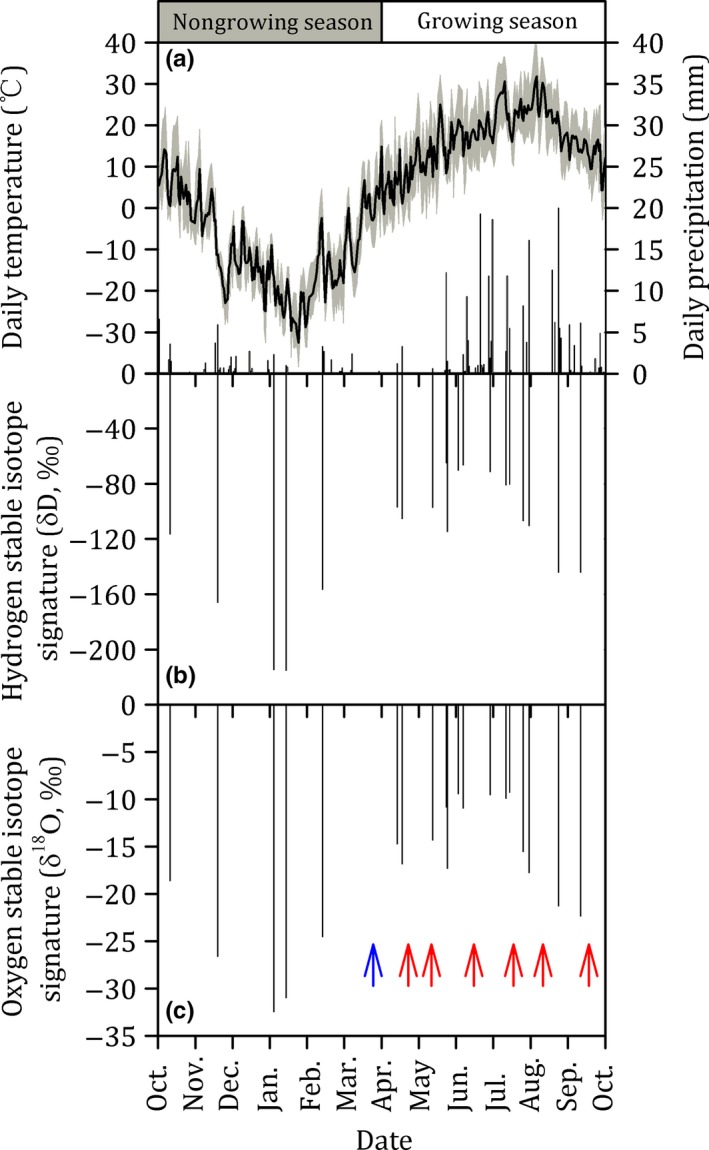
Daily maximum, minimum, and mean air temperature (lines), precipitation (bars) (a), hydrogen stable isotope signature (δ*D*, ‰) (b), and oxygen stable isotope signature (δ^18^O, ‰) (c) in precipitation from 1 October 2015 to 30 September 2016 in the temperate steppe in Inner Mongolia, China. The nongrowing season is observed to be between early October and late March of the following year. The growing season is from early April to late September. The blue arrow indicates the day when all snow had melted, that is, the snow‐free date. The red arrows mark the days when soil and plant samples were collected for hydrogen and oxygen stable isotopes analysis

The δ*D*_snowfall_ value during the nongrowing season (i.e., from October 1 to March 31 of the following year) was −173.964 ± 10.041‰ and ranged from −217.690 to −114.522‰. In comparison, the δ*D*_rainfall_ value during the growing season (i.e., from April 1 to September 30) was −97.342 ± 3.470‰ and ranged from −146.430 to −64.218‰ (Figure 1).

The δ^18^O_snowfall_ value during the nongrowing season was −26.671 ± 1.321‰ and ranged from −33.079 to −18.298‰. In comparison, the δ^18^O_rainfall_ value during the growing season was −14.511 ± 0.597‰ and ranged from −22.792 to −8.642‰ (Figure 1).

### 3.2 Contributions of snowfall versus rainfall to soil water

**Figure 2:**
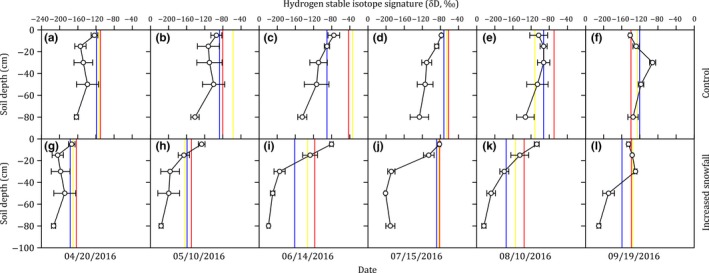
Hydrogen stable isotope signature (δ*D*, ‰) in soil water profile (circles) and in plant water of species with different rooting depths (red lines: shallow‐rooted species, *Stipa grandis *P. Smirn.; yellow lines: medium‐rooted species, *Agropyron cristatum* (L.) Garrtn.; blue lines: deep‐rooted species, *Artemisia frigida* Willd.) in control (top panels) and increased snowfall plots (bottom panels) during the growing season (early April to late September). Values are mean ± *SE* (*N* = 3)

**Figure 3:**
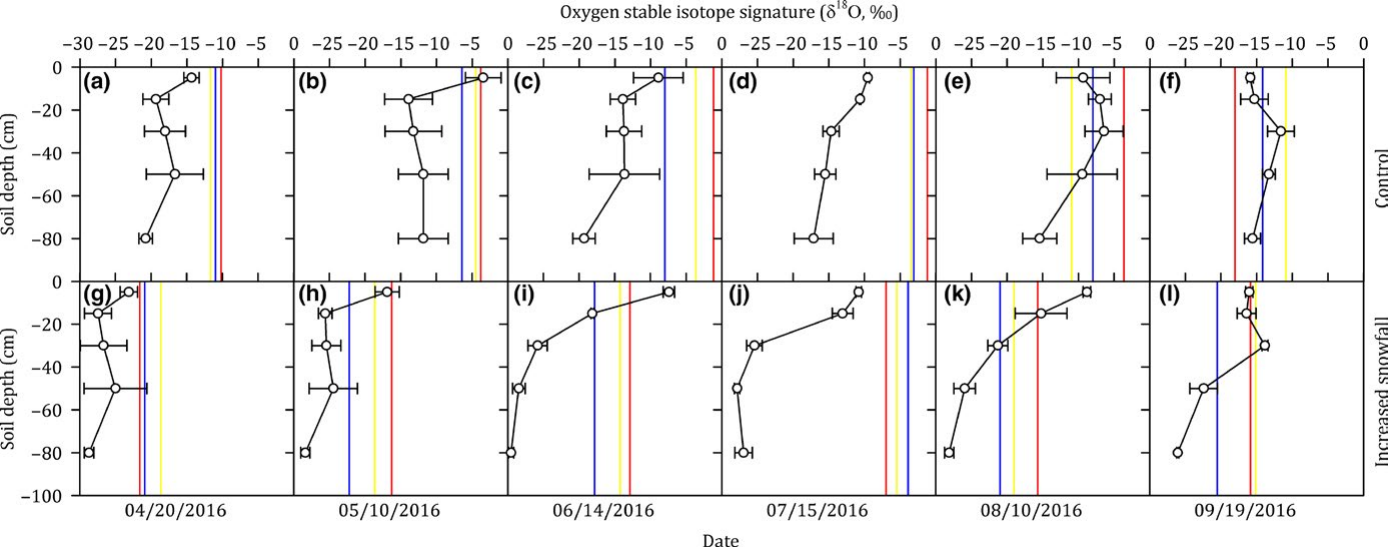
Oxygen stable isotope signature (δ^18^O, ‰) in soil water profile (circles) and in plant water of species with different rooting depths (red lines: shallow‐rooted species, *Stipa grandis *P. Smirn.; yellow lines: medium‐rooted species, *Agropyron cristatum* (L.) Garrtn.; blue lines: deep‐rooted species, *Artemisia frigida* Willd.) in control (top panels) and increased snowfall plots (bottom panels) during the growing season (early April to late September). Values are mean ± *SE* (*N* = 3)

The δ*D*_soil_ value was significantly affected by the treatments (*p* < 0.001) and soil depths (*p* < 0.001) (Supporting Information Table S1). Compared with the control plots (−115.891 ± 3.389‰), the δ*D*_soil_ in the increased snowfall plots (−171.842 ± 4.751‰) significantly decreased (*p* < 0.001, Figure 2; Supporting Information Table S1). The δ*D*_soil_ in the topsoil (0–10 cm, −110.872 ± 5.499‰) was less negative than that in the subsoil (60–100 cm, −176.444 ± 6.818‰) (Figure 2). The δ*D*_soil_ increased slightly from −158.396 ± 5.903‰ in the early growing season (April–May) to a peak of −132.456 ± 6.677‰ in the middle growing season (June–July) and was followed by a decline to −140.748 ± 5.611‰ in the late growing season (August–September) (Figure 2). The results of δ^18^O_soil_ showed the similar pattern with δ*D*_soil _(Figure 3; Supporting Information Table S1).

**Figure 4:**
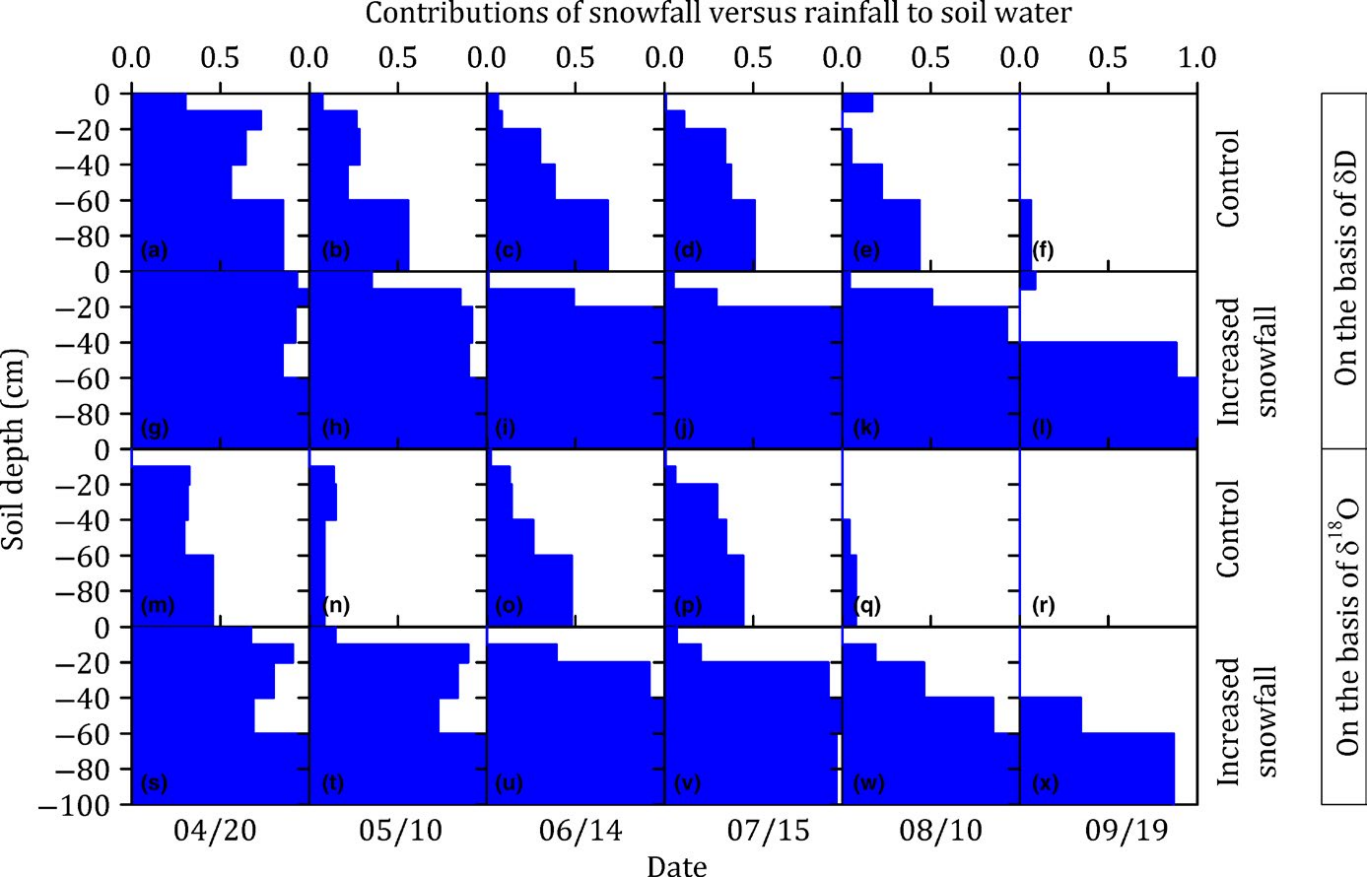
The contributions of snowfall (blue) versus rainfall (white) to soil water at different soil depths (0–100 cm) in the control (a–f and m–r) and increased snowfall plots (g–l and s–x) during the growing season (early April to late September) in 2016 in the temperate steppe in Inner Mongolia, China

Snowfall versus rainfall accounted for 49% and 51%, respectively, of the water source for soil on the basis of δ*D* analysis and accounted for 37% and 63%, respectively, on the basis of δ^18^O analysis, when the data of treatments, soil depths, and sampling dates were all pooled (Figure 4). The contributions of snowfall to soil water were significantly affected by the treatments (both *p* < 0.001 based on δ*D* and δ^18^O analysis) and soil depths (both *p* < 0.001 based on δ*D* and δ^18^O analysis) (Supporting Information Table S1).

### 3.3 Contributions of snowfall versus rainfall to plant water

The δ*D*_plant_ value was significantly affected by the treatments (*p* < 0.001) and species (*p* = 0.049) (Supporting Information Table S2). Compared with the control plots (−90.573 ± 4.512‰), δ*D*_plant_ in the increased snowfall plots (−142.379 ± 4.708‰) significantly decreased (*p* < 0.001, Figure 2; Supporting Information Table S2). The δ*D*_plant_ values in the shallow‐rooted species (i.e., *Stipa grandis *P. Smirn., −108.344 ± 6.697‰) were less negative than that in the deep‐rooted species (i.e., *Artemisia frigida* Willd., −126.986 ± 7.127‰) (Figure 2). The δ*D*_plant_ values increased from −129.619 ± 6.984‰ in the early growing season (April–May) to a peak of −88.363 ± 6.124‰ in the middle growing season (June–July) before declining to −131.446 ± 5.824‰ in the late growing season (August–September) (Figure 2). The results of δ^18^O_plant_ showed the similar pattern with δ*D*_plant_ (Figure 3; Supporting Information Table S2).

**Figure 5:**
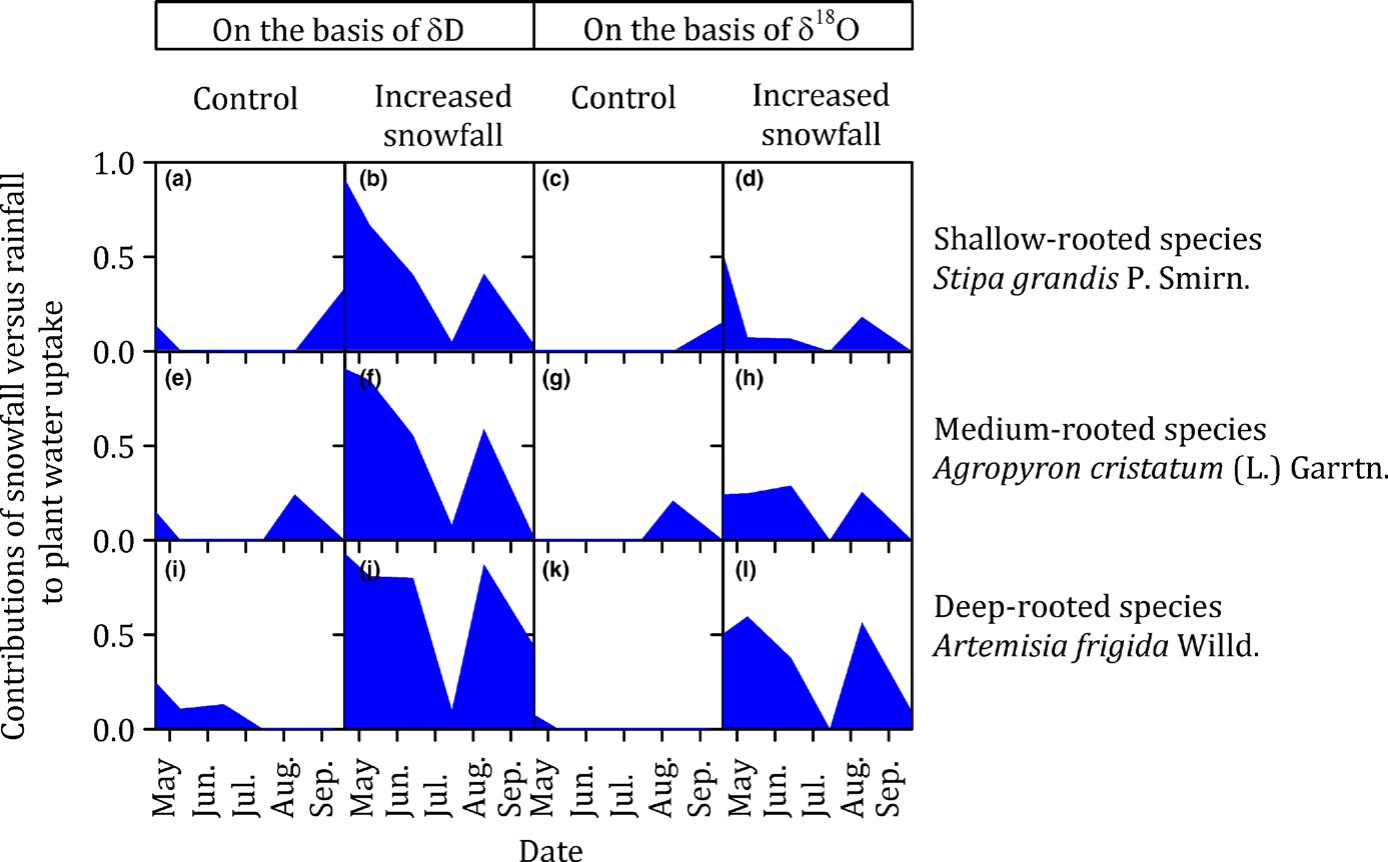
The contributions of snowfall (blue) versus rainfall (white) to plant water uptake of species with different rooting depths (i.e., shallow‐rooted species, *Stipa grandis *P. Smirn. (a–d); medium‐rooted species, *Agropyron cristatum* (L.) Garrtn. (e–h); deep‐rooted species, *Artemisia frigida* Willd. (i–l)) in the control and increased snowfall plots during the growing season (early April to late September) in 2016 in the temperate steppe in Inner Mongolia, China

Snowfall versus rainfall accounted for 30% and 70%, respectively, of the water source for plants, on the basis of δ*D* analysis, and accounted for 12% and 88%, respectively, on the basis of δ^18^O analysis, when the data of treatments, species, and sampling dates were all pooled (Figure 5). The contributions of snowfall to plant water were significantly affected by the treatments (*p* < 0.001 based on δ*D* analysis, *p* = 0.002 based on δ^18^O analysis), but not by the species (*p* = 0.240 based on δ*D*, *p* = 0.263 based on δ^18^O) (Supporting Information Table S2).

### 3.4 Main depth of root water uptake

**Figure 6:**
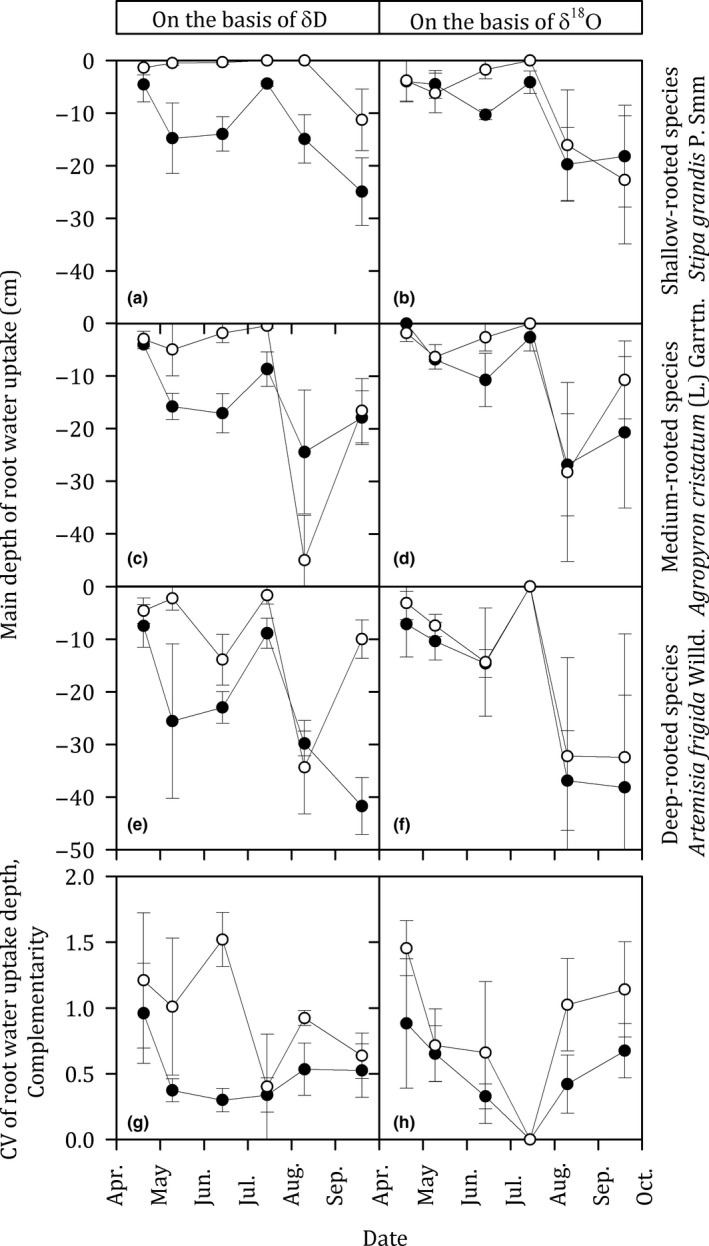
The main depth of root water uptake for species with different rooting depths (i.e., shallow‐rooted species, *Stipa grandis *P. Smirn. (a, b); medium‐rooted species, *Agropyron cristatum* (L.) Garrtn. (c, d); deep‐rooted species, *Artemisia frigida* Willd. (e, f)); and the coefficient of variation (CV) (g, h) for the main depth of root water uptake among species in the control (open circles) and increased snowfall plots (filled circles) during the growing season (early April to late September)

BGB was significantly increased by increased snowfall treatments (*p* = 0.020, Supporting Information Table S3). On the basis of δ*D* analysis, the main depth of root water uptake was significantly affected by treatments (*p* = 0.001) and species (*p* = 0.006) (Figure 6; Supporting Information Table S2). The CV of the main depth of root water uptake among species was significantly decreased by increased snowfall treatments (*p* = 0.016) (Figure 6g, Supporting Information Table S3).

On the basis of δ^18^O analysis, the main depth of root water uptake was marginally affected by the species (*p* = 0.106), but not by increased snowfall treatments (*p* = 0.377) (Figure 6; Supporting Information Table S2). The CV of the main depth of root water uptake among species was marginally decreased by increased snowfall treatments (*p* = 0.087) (Figure 6h; Supporting Information Table S3).

## 4 DISCUSSION

### 4.1 Snowfall contribution to plant water uptake continued throughout the growing season

Our study reported that the snowfall contribution to plant water uptake continued throughout the growing season. It has been proposed that snowfall contribute to plant water uptake only in the early growing season and rainfall contribute to plant water uptake in the middle and late growing season (Hu et al., 2013). However, in our study, the snowfall contribution to plant water uptake continued throughout the entire growing season and was detectable even in the late growing season (Figure 5). In this semiarid region, winter snowfall mainly recharges the subsoil, while the topsoil is mainly recharged by summer rainfall (Figure 4). Consequently, the nongrowing season snowfall could contribute to plant water uptake in the growing season (Figure 5), when the topsoil water was largely depleted by evapotranspiration.

Snowfall was an important water source for plants grown in semiarid regions (Supporting Information Figures S2). Our study showed that snowfall accounted for 30% of the water source for plants, on the basis of δ*D* analysis, and accounted for 12% on the basis of δ^18^O analysis, when the data of treatments, species, and sampling dates were all pooled (Figure 5). Snowfall represented 26% of the annual precipitation in current study, when the data of control and increased snowfall plots were all pooled. A previous study, conducted in the same region, had also reported that snowfall accounted for 15% of the water source for plants, in the context that snowfall represented 11% of the annual precipitation (Yang et al., 2011). Therefore, our observations suggested that the snowfall contribution to plant water uptake was roughly equal to the ratio of snowfall to precipitation in this semiarid region.

### 4.2 Increased snowfall weakened complementarity of water use by different plant functional groups

Water use partitioning between shallow‐rooted and deep‐rooted plants was found in our study. For example, *Stipa grandis *P. Smirn., that is, a shallow‐rooted species, always depended on topsoil water (Figure 6). However, *Artemisia frigida* Willd., that is, a deep‐rooted species, mainly accessed subsoil water (Figure 6). Root architecture, that is, the distribution of roots in the soil profile, may be responsible for the pattern of water use partitioning between the topsoil and the subsoil among the different plant functional groups (Nippert & Holdo, 2015). In addition, root plasticity, that is, the ability of plants to shift water uptake among different water sources (Ehleringer & Dawson, 1992), was also found in our study (Figure 6). For example, plants used topsoil water in July when rainfall was high and used subsoil water in August when topsoil water was depleted (Figure 6).

Our study based on hydrogen and oxygen stable isotopes analysis showed that increased snowfall weakened complementarity of summer water use by different plant functional groups. Under an increased snowfall regime, the CV of the main depth of root water uptake among species, which represented complementarity of water use (Kühsel & Blüthgen, 2015), was significantly decreased (Figure 6g). It has been proposed that complementarity might be achieved through among‐species differences in resource uptake in terms of time and space (von Felten et al., 2009; Kahmen, Renker, Unsicker, & Buchmann, 2006). Therefore, the depressed complementarity of water use among different plant functional groups in current study was thought to result from an increased similarity in rooting depths among species (Bachmann et al., 2015).

**Figure 7:**
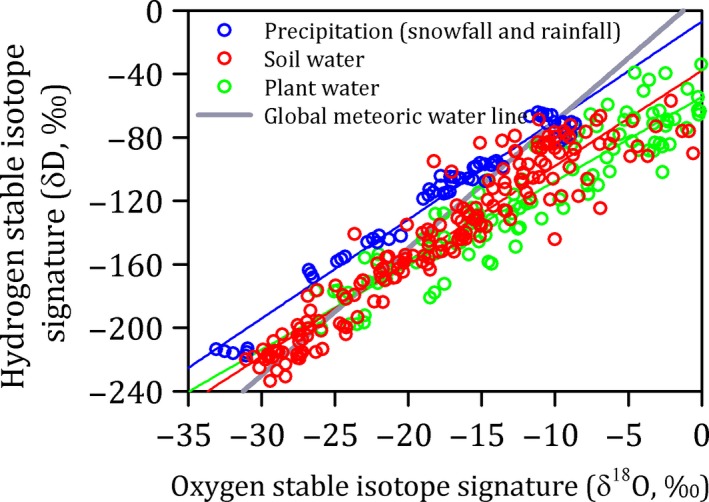
δ^18^O‐δ*D* relationships of water samples in the temperate steppe in Inner Mongolia

There were three issues worthy of note. First, the residual soil water within 0–100 cm before rain and snow fell was about 50 mm and accounted for 18% of the annual precipitation according to a previous study in the same study area (Yang et al., 2011). Our assumption of the two‐source linear mixing models ignored the effects of the residual soil water, which would overestimate the importance of snowfall. Second, the evaporative enrichment of isotopes was evaluated through fitting δ*D*–δ^18^O relationships of water samples. The relationships between δ*D* and δ^18^O were δ*D* = 6.26δ^18^O − 7.10 (*R* ^2^ = 0.97), δ*D* = 6.00δ^18^O‐37.90 (*R* ^2^ = 0.90), and δ*D* = 5.31δ^18^O − 54.58 (*R* ^2^ = 0.86), respectively, for precipitation, soil water, and plant water (Figure 7). Their slopes and intercepts were less than the globe meteoric water line (GMLW: δ*D* = 8.00δ^18^O + 10.00), which reflected the evaporation enrichment of isotopes in precipitation, soil water, and plant water. As such, we identified that our assumption of the two‐source linear mixing models ignored the effects of the evaporation enrichment of isotopes, which has uncertainty. Third, the small sample sizes and temporal period (1 year) limited the extrapolation of our findings to the greater Mongolian grassland region and other steppe ecosystems.

In conclusion, three aspects of our study distinguished it from the few previous studies about the effects of snowfall on ecosystem functioning. First, our study demonstrated that the snowfall contribution to plant water continued throughout the growing season and was detectable even in the late growing season. Second, our study quantified the snowfall contribution to plant water uptake, which was roughly equal to the ratio of snowfall to precipitation. Third, our study provided the evidence based on hydrogen and oxygen stable isotopes analysis that increased snowfall weakened complementarity of summer water use by different plant functional groups.

## CONFLICT OF INTEREST

The authors have no conflict of interests to declare.

## AUTHOR CONTRIBUTIONS

Yonggang Chi (YC) designed the research. YC, Lei Zhou (LZ), and Shuxia Zheng (SZ) conducted the field sampling. YC and Qingpeng Yang (QY) performed the sample analyses. YC, LZ, and Shao‐peng Li (SL) analyzed the data. YC, LZ, QY, SL, and SZ wrote the manuscript. All authors approved the final version of the manuscript.

## DATA AVAILABILITY

Data available from the Dryad Digital Repository: https://doi.org/10.5061/dryad.6fp82p0.
