# Supplementary material for: Increased snowfall weakens complementarity of summer water use by different plant functional groups
Source: Ecol Evol. 2019 Mar 15;9(7):4264–74. doi: 10.1002/ece3.5058 (PMC6468069; doi:10.1002/ece3.5058)
Supplement: Supplementary file 1 [file ECE3-9-4264-s001.docx]

**Supporting Information**

**Table S1** Statistical results of repeat-measurement ANOVA on the effects of treatment, soil depth, sampling date, and their interactions on the hydrogen stable isotope signature of the soil water (δD_soil_), the oxygen stable isotope signature of the soil water (δ^18^O_soil_), snowfall contribution to soil water on the basis of δD analysis, snowfall contribution to soil water on the basis of δ^18^O analysis, and soil water content (SWC).

|  | δD_soil_ | | |  | δ^18^O_soil_ | | |  | Snowfall contribution to soil water on the basis of δD analysis | | |  | Snowfall contribution to soil water on the basis of δ^18^O analysis | | |  | SWC | | |
| --- | --- | --- | --- | --- | --- | --- | --- | --- | --- | --- | --- | --- | --- | --- | --- | --- | --- | --- | --- |
|  | d.f. | *F* | *P* |  | d.f. | *F* | *P* |  | d.f. | *F* | *P* |  | d.f. | *F* | *P* |  | d.f. | *F* | *P* |
| Treatments | 1 | 122.93 | **0.000** |  | 1 | 134.52 | **0.000** |  | 1 | 143.31 | **0.000** |  | 1 | 144.14 | **0.000** |  | 1 | 13.95 | **0.001** |
| Soil depths | 4 | 20.02 | **0.000** |  | 4 | 21.74 | **0.000** |  | 4 | 32.93 | **0.000** |  | 4 | 23.11 | **0.000** |  | 4 | 49.67 | **0.000** |
| Treatments×depths | 4 | 6.31 | **0.002** |  | 4 | 4.98 | **0.006** |  | 4 | 6.09 | **0.002** |  | 4 | 7.14 | **0.001** |  | 4 | 0.80 | 0.540 |
| Dates | 5 | 13.34 | **0.000** |  | 5 | 13.55 | **0.000** |  | 5 | 21.03 | **0.000** |  | 5 | 25.38 | **0.000** |  | 5 | 50.28 | **0.000** |
| Dates×treatments | 5 | 2.15 | 0.065 |  | 5 | 4.82 | **0.001** |  | 5 | 1.02 | 0.412 |  | 5 | 4.73 | **0.001** |  | 5 | 1.06 | 0.390 |
| Dates×depths | 20 | 4.08 | **0.000** |  | 20 | 3.81 | **0.000** |  | 20 | 2.37 | **0.003** |  | 20 | 3.85 | **0.000** |  | 20 | 34.44 | **0.000** |
| Dates×treatments×depths | 20 | 0.94 | 0.538 |  | 20 | 1.42 | 0.130 |  | 20 | 2.33 | **0.003** |  | 20 | 3.09 | **0.000** |  | 20 | 0.67 | 0.848 |

**Table S2** Statistical results of repeat-measurement ANOVA on the effects of treatment, species, sampling date, and their interactions on the hydrogen stable isotope signature of the plant water (δD_plant_), the oxygen stable isotope signature of the plant water (δ^18^O_plant_), snowfall contribution to plant water uptake on the basis of δD analysis, snowfall contribution to plant water uptake on the basis of δ^18^O analysis, main depth of root water uptake on the basis of δD analysis, main depth of root water uptake on the basis of δ^18^O analysis.

|  | δD_plant_ | | |  | δ^18^O_plant_ | | |  | Snowfall contribution to plant water uptake on the basis of δD analysis | | |  | Snowfall contribution to plant water uptake on the basis of δ^18^O analysis | | |  | Main depth of root water uptake on the basis of δD analysis | | |  | Main depth of root water uptake on the basis of δ^18^O analysis | | |
| --- | --- | --- | --- | --- | --- | --- | --- | --- | --- | --- | --- | --- | --- | --- | --- | --- | --- | --- | --- | --- | --- | --- | --- |
|  | d.f. | *F* | *P* |  | d.f. | *F* | *P* |  | d.f. | *F* | *P* |  | d.f. | *F* | *P* |  | d.f. | *F* | *P* |  | d.f. | *F* | *P* |
| Treatments | 1 | 85.93 | **0.000** |  | 1 | 93.62 | **0.000** |  | 1 | 58.66 | **0.000** |  | 1 | 15.41 | **0.002** |  | 1 | 19.04 | **0.001** |  | 1 | 0.84 | 0.377 |
| Species | 2 | 3.91 | **0.049** |  | 2 | 2.77 | 0.103 |  | 2 | 1.61 | 0.240 |  | 2 | 1.49 | 0.263 |  | 2 | 8.13 | **0.006** |  | 2 | 2.72 | 0.106 |
| Treatments×species | 2 | 0.17 | 0.846 |  | 2 | 0.27 | 0.772 |  | 2 | 1.47 | 0.270 |  | 2 | 2.06 | 0.170 |  | 2 | 2.21 | 0.153 |  | 2 | 0.02 | 0.978 |
| Dates | 5 | 38.12 | **0.000** |  | 5 | 20.36 | **0.000** |  | 5 | 17.05 | **0.000** |  | 5 | 3.76 | **0.005** |  | 5 | 17.95 | **0.000** |  | 5 | 9.47 | **0.000** |
| Dates×treatments | 5 | 14.56 | **0.000** |  | 5 | 5.98 | **0.000** |  | 5 | 12.05 | **0.000** |  | 5 | 3.47 | **0.008** |  | 5 | 3.85 | **0.004** |  | 5 | 0.07 | 0.996 |
| Dates×species | 10 | 2.11 | **0.037** |  | 10 | 1.38 | 0.210 |  | 10 | 0.96 | 0.486 |  | 10 | 0.85 | 0.586 |  | 10 | 2.77 | **0.007** |  | 10 | 0.60 | 0.806 |
| Dates×treatments×species | 10 | 0.88 | 0.556 |  | 10 | 0.72 | 0.704 |  | 10 | 1.36 | 0.223 |  | 10 | 0.87 | 0.562 |  | 10 | 1.93 | 0.059 |  | 10 | 0.14 | 0.999 |

**Table S3** The effects of increased snowfall on above-ground biomass (AGB), below-ground biomass (BGB), and the coefficient of variation (CV) for the main depth of root water uptake among species.

| Treatments | AGB  (g/m^2^) | BGB  (0-10 cm, g/m^2^) | BGB  (10-20 cm, g/m^2^) | BGB  (20-40 cm, g/m^2^) | BGB  (40-60 cm, g/m^2^) | BGB  (60-100 cm, g/m^2^) | BGB  (0-100 cm, g/m^2^) | Coefficient of variation (CV) for the main depth of root water uptake among species on the basis of δD analysis | Coefficient of variation (CV) for the main depth of root water uptake among species on the basis of δ^18^O analysis |
| --- | --- | --- | --- | --- | --- | --- | --- | --- | --- |
| Control | 184.05±7.96 | 218.04±9.52 | 148.40±12.15 | 128.89±7.63 | 98.46±10.44 | 84.37±16.30 | 678.17±17.10 | 0.95±0.15 | 0.83±0.16 |
| Increased snowfall | 281.80±25.42 | 464.42±23.53 | 257.25±33.69 | 128.73±23.97 | 118.52±10.21 | 105.03±31.47 | 1073.96±104.92 | 0.50±0.09 | 0.47±0.11 |
| d.f. | 1 | 1 | 1 | 1 | 1 | 1 | 1 | 1 | 1 |
| *F* | 13.46 | 94.30 | 9.24 | 0.00 | 1.89 | 0.34 | 13.86 | 6.47 | 3.09 |
| *P* | **0.021** | **0.001** | **0.038** | 0.999 | 0.24 | 0.592 | **0.020** | **0.016** | 0.087 |


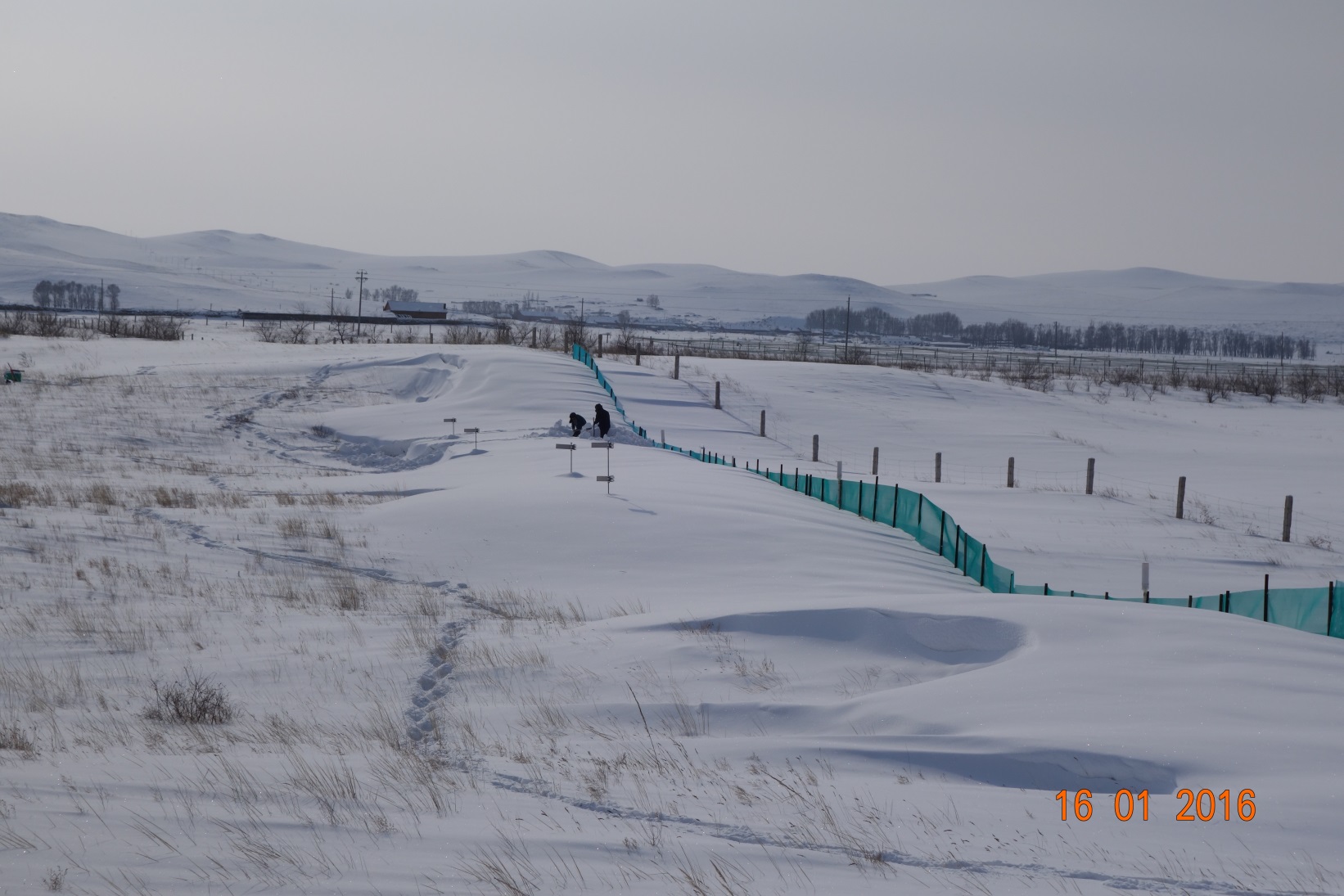


**Control**

**Increased snowfall**


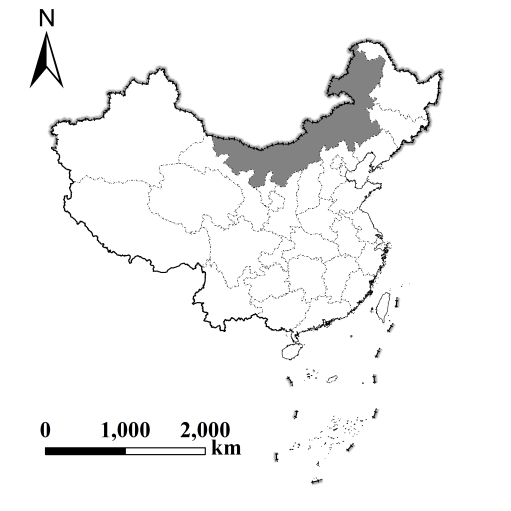


**Figure S1** Layout of the experiment plots in the Inner Mongolia grasslands, China. The field experiment includes two snow depth regimes (i.e., the control and the increased snowfall) with three replicates for each treatment.





**Figure S2** Soil water content (g/g) at different soil depths (0-100 cm) in the control (open circles) and increased snowfall plots (filled circles) during the growing season (early April to late September) in 2016 in the temperate steppe in Inner Mongolia, China. Values are mean ± SE (N=3).
